# Plant Recycling for Molecular Biofarming to Produce Recombinant Anti-Cancer mAb

**DOI:** 10.3389/fpls.2016.01037

**Published:** 2016-07-18

**Authors:** Deuk-Su Kim, Ilchan Song, Jinhee Kim, Do-Sun Kim, Kisung Ko

**Affiliations:** ^1^Department of Medicine, College of Medicine, Chung-Ang UniversitySeoul, South Korea; ^2^Vegetable Research Division, National Institute of Horticultural and Herbal Science, Rural Development AdministrationWanju-gun, South Korea

**Keywords:** plant product system, axillary bud, biomass, recycling plant, anti colorectal monoclonal antibody

## Abstract

The expression and glycosylation patterns of anti-colorectal cancer therapeutic monoclonal antibody (mAb) CO17-1A recognizing the tumor-associated antigen GA733-2, expressed in human colorectal carcinoma cells, were observed in the leaf and stem tissues of primary (0 cycle), secondary (1 cycle), and tertiary (2 cycle) growths of seedlings obtained from the stem cut of T_2_ plants. The bottom portion of the stem of T_2_ seedlings was cut to induce the 1 cycle shoot growth, which was again cut to induce the 2 cycle shoot growth. In the 1 and 2 cycle growths, the periods for floral organ formation (35 days) was shorter than that (100 days) for the 0 cycle growth. The genes of heavy and light chains of mAb CO17-1A existed at the top, middle, and basal portions of the leaves and stem obtained from the 0, 1, and 2 cycle plants. The protein levels in the leaves and stem tissues from the 1 and 2 cycles were similar to those in the tissues from the 0 cycle. The glycosylation level and pattern in the leaf and stem did not alter dramatically over the different cycles. Surface plasmon resonance (SPR) confirmed that mAbs CO17-1A obtained from leaf and stem tissues of the 0, 1, and 2 cycles had similar binding affinity for the GA733-2 antigen. These data suggest that the shoot growth by bottom stem cutting is applicable to speed up the growth of plant biomass expressing anti-colorectal cancer mAb without variation of expression, glycosylation, and functionality.

## Background

Plants are well recognized as alternative hosts for production of highly valuable recombinant proteins, such as antibodies, vaccines, human blood products, hormones, and growth regulators (Fernandez-San Millan et al., 2003; Rigano and Walmsley, 2005; Schillberg et al., 2013). They offer mass production and safety advantages, with ease of seed storage, compared to the microbial and animal cell-based systems (Twyman et al., 2003; Fischer et al., 2013; Shanmugaraj and Ramalingam, 2014). Plant cultivation can easily be modulated in response to the demand for the recombinant protein by controlling the number of seeds sown (Fischer and Emans, 2000; Leckie and Stewart, 2011). The speed of obtaining plant biomass is essential in the application to save time for production of recombinant proteins. In general, however, more than 14 weeks are required for the development of a fully grown tobacco plant from the time of sowing of its seeds (Lim et al., 2015). A plant-based recombinant protein production system might have drawbacks, such as relatively long cultivation period for obtaining full biomass from the seeds, especially when there is time-constraint for use of the field for cultivation of such plants. Therefore, rapid enhancement of the plant biomass is imperative for increasing the efficiency of plant-based systems for production of recombinant proteins.

Physiological mechanisms responsible for rapid growth of lateral shoots, without a corresponding increase in root growth, can promote biomass accumulation (Vysotskaya, 2005). Auxin, an important hormone of shoots, in particular, regulates apical dominance (Cline et al., 1997; Teale et al., 2006). The lower lateral buds can be induced by cutting the terminal bud to remove apical dominance (Skoog and Thimann, 1934; Leyser, 2003; Umehara et al., 2008). Moreover, the speed of plant shoot growth could be enhanced by lowering the shoot/root ratio (Vysotskaya, 2005), to overcome the space and time limitations. The cutting of lower branches could be a strategy for plant recycling system to speed up the biomass production without the need for further sowing of seeds. In this study, the effect of cutting the stem to remove apical dominance on shoot growth rate was determined. Furthermore, the expression, glycosylation, and function of a recombinant anti-colorectal cancer monoclonal antibody (mAb) CO17-1A, expressed in the newly induced shoots was determined to confirm whether secondary shoot growth would be applicable as a plant recycling system to obtain increased biomass for enhanced production of recombinant anti-cancer mAb proteins.

## Materials and Methods

### Plant Material and Cultivation

**FIGURE 1:**
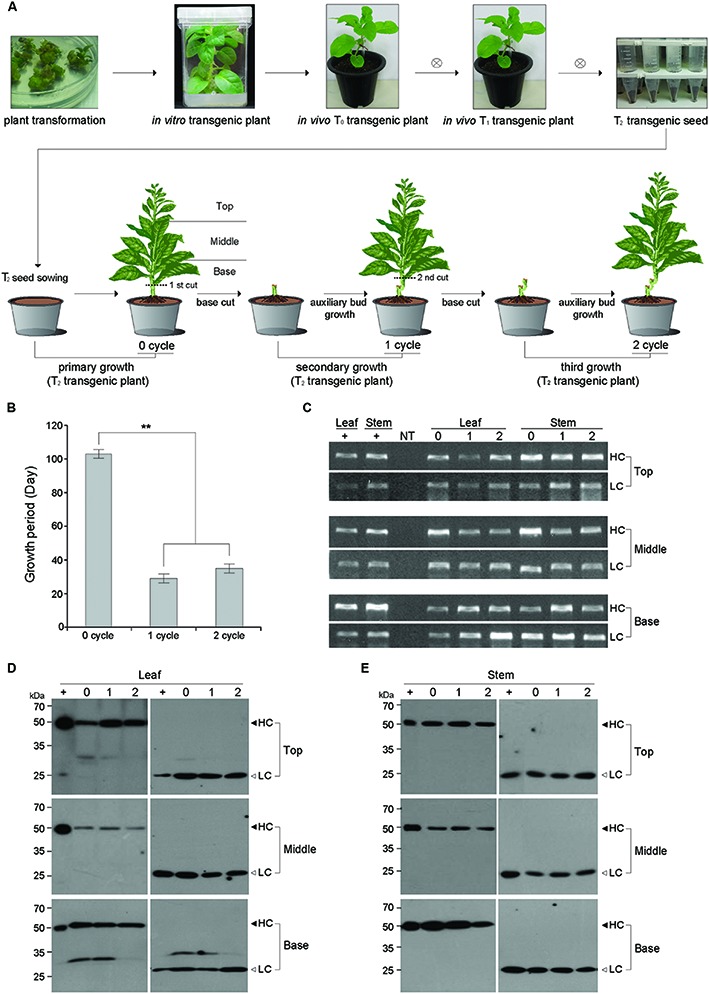
**Schematic diagram for the primary (0 cycle), secondary (1 cycle), and tertiary (2 cycle) growth of plants expressing the anti-colorectal cancer mAb CO17-1A. (A)** Schematic diagram showing primary (0 cycle), secondary (1 cycle), and tertiary (2 cycle) growths from T_0_ transgenic plants. The base stem of 0 cycle plant was cut to induce axillary buds for secondary plant growth (1 cycle), and the base stem of the 1 cycle plant was cut for the tertiary plant growth (2 cycle). T, top of the whole plant; M, middle of the whole plant; B, base of the whole plant. **(B)** Comparison of plant growth period for flowering in 0, 1, and 2 cycles. The growth of 0 cycle plants was compared with 1 and 2 cycle plants obtained from base stem cutting. The asterisks indicate statistically significant differences (^∗∗^*p* < 0.01). **(C)** PCR analysis to confirm the existence of HC and LC genes in top, middle, and basal portions of both the leaves and stems of transgenic plant (0, 1, and 2 cycles). The genomic DNA fragments of mAb^P^ CO17-1A were amplified and electrophoretically separated on a 1% agarose gel. NT, non-transgenic plant; HC, heavy chain of mAb^P^ CO17-1A; LC, light chain of mAb^P^ CO17-1A. **(D,E)** Western blot analysis to confirm the mAb^P^ CO17-1A HC and LC expression in the leaves and stems of transgenic plants through 0, 1, and 2 cycles. The bands for HC (50 kDa) and LC (25 kDa) were detected with horse radish peroxidase-conjugated goat anti-mouse Fc and goat anti-mouse F(ab)′_2_-specific antibody, respectively. +, purified mAb CO17-1A from plant (So et al., 2012); Top, top portion of plant; Middle, middle portion of plant; Base, basal portion of plant **(A)**.

Forty seeds of transgenic tobacco T_2_ plants, with plant-derived anti-colorectal cancer mAb (mAb^P^ CO17-1A) production capability (So et al., 2012), were sown in pots (18.5 cm × 18.5 cm × 14.5 cm) filled with steam-sterilized commercial soil mixture (Sun Gro Horticulture, Agawam, MA; **Figure 1A**), respectively. Forty seedlings were grown in greenhouse under simulated natural light conditions with an average 12 h light/12 h dark photoperiod. The growth of plants was measured immediately before flowering. The lower branch of the primary plant seedling (0 cycle) was cut to induce axillary buds for growth of lateral branches (1 cycle) on the remnant 10 cm long base stem (**Figure 1A**). The base stem of the 0 cycle plant in the pot was maintained to induce the 1 cycle for the growth of lateral branches until the appearance of inflorescence on the shoot obtained from the axillary bud (**Figure 1A**). The growth of tertiary (2 cycle) shoots was induced from the cut stem of the fully grown l cycle plant (**Figure 1A**).

### Polymerase Chain Reaction (PCR) Amplification from Genomic DNA of Leaf and Stem in 0, 1, and 2 Cycle Plants

Genomic DNA was isolated from approximately 100 mg of leaf and stem tissues from the plants (0, 1, and 2 cycles) using DNeasy kit (Qiagen, Hilden, Germany), according to the manufacturer’s recommendations. The extracted DNA was amplified by polymerase chain reaction (PCR) to confirm the presence of genes for mAb CO17-1A heavy chain (HC; 1,471 bp) and light chain (LC; 764 bp), by using the following forward and reverse primers: HC forward primer, 5′-GCGAATTCATGGAA TGGAGCAGAGTCTTTAT C-3′; HC reverse primer, 5′-GATTA ATCGATTTTACCCGGAGTCCG-3′; LC forward primer, 5′-GC CTCG AGATGGGCATCAAGATGGAATCACAG-3′; LC reverse primer, 5′-GAGGTACCCTAACACTCATTCCTGTTGAAGCTC-3′.

### Western Blot Analysis

Eighty milligram of fresh leaves and stems (from top, middle, and basal portions of plants) was crushed by cryo-milling to extract the total soluble proteins. The homogenized plant samples were mixed with 280 μL of sample buffer (1 M Tris-HCl, 50% glycerol, 10% SDS, 5% 2-mercaptoethanol, 0.1% bromophenol blue), and the homogenates were loaded on a sodium dodecyl sulfate polyacrylamide gel. The electrophoresed proteins were transferred on to a nitrocellulose membrane (Millipore Corp., Billerica, MA, USA), which was blocked with 5% skimmed milk (Sigma, St. Louis, MO, USA), prepared in 1× phosphate-buffered saline (PBS), for 2 h. The blot was subsequently probed with goat anti-murine IgG Fcγ and anti-murine IgG F(ab)′_2_, which recognize the HC and LC of mAb CO17-1A, respectively. The purified mAb^P^ CO17-1A was used as a positive control (Ko et al., 2005).

### Purification of Recombinant mAb^P^ CO17-1AK from Leaf and Stem of Plant from Each Cycle

For purification of mAb^P^ CO17-1AK, the leaves and stem from the tobacco plants of the 0, 1, and 2 cycles were homogenized on ice in the extraction buffer (37.5 mM Tris-HCl pH 7.5, 50 mM NaCl, 15 mM EDTA, 75 mM sodium citrate, and 0.2% sodium thiosulfate) using a blender. After centrifugation at 8,800 × *g* for 30 min at 4°C, the supernatant was filtered through a Miracloth (Biosciences, La Jolla, CA, USA), and its pH was adjusted to 5.1 with acetic acid. The supernatant was further centrifuged at 10,200 × *g* for 30 min. The pH of the supernatant, thus obtained, was adjusted to neutral by addition of 3 M Tris-HCl. The total soluble protein was precipitated with ammonium sulfate after overnight incubation in a cold room followed by centrifugation at 4°C for 30 min. The pellet was resuspended in one-tenth of the starting volume of extraction buffer, and the obtained solution was centrifuged at 10,200 × *g* for 30 min at 4°C (Park et al., 2015). The mAb^P^ CO17-1A protein was purified using protein A Sepharose 4 Fast Flow (GE Healthcare, Sweden, NJ, USA), according to the manufacturer’s recommendations. The mAb^P^ CO17-1A protein was dialyzed against 1× PBS (pH 7.4). The protein concentration was determined using a Nano-drop (Biotek, Highland, VT, USA) and the purified protein was visualized by SDS-PAGE. Aliquots of the purified protein were stored at -80°C for further studies.

### Glycan Analysis

The purified mAb^P^ CO17-1A protein samples were treated twice with 1 μL pepsin in an incubator at 37°C for 16 h to digest the protein into glycopeptides. The glycopeptides were collected using a C18 Sep-Pak cartridge (Waters, Lexington, MA, USA). The *N*-glycosidase (PNGase) A glycan enzyme was added to the collected glycopeptides to release the *N*-glycans, and the mixtures were incubated overnight at 37°C. The released *N*-glycans were purified from the samples by using a graphitized carbon resin from Carbograph (Alltech, Lexington, MA, USA). The purified glycans were 2-aminobenzamide (2-AB)-labeled using previously described methods (Bigge et al., 1995). The 2-AB-labeled glycans were separated on a TSK amide-80 column (5 μm, 4.6 mm × 250 mm; Tosoh Bioscience, Prussia, PA, USA) using a high performance liquid chromatography (HPLC) system with a fluorescence detector (330 nm excitation and 425 nm emission; Lim et al., 2015). The separation of the labeled glycans was achieved at a flow rate of 1.0 mL/min using a mixture of solvent A (100% acetonitrile) and solvent B (50 mM ammonium formate, pH 4.4). After the column was equilibrated using 30% solvent B, the sample was injected and then eluted by a linear gradient to 45% of solvent B for 60 min. HPLC analysis was repeated more than three times.

### Surface Plasmon Resonance Analysis

The surface plasmon resonance (SPR) analysis was performed to confirm the affinity of mAb^P^ CO17-1A to GA733 antigen using a commercially available GLC chip on an XPR36 surface instrument (Bio-Rad, Hercules, CA, USA). The GA733 protein was immobilized on a GLC chip, and an acidic buffer at pH 6.0 was allowed to flow over the biochip surface at a rate of 50 μL/min. One microgram of the purified mAb^P^ CO17-1A from leaf and stem (0, 1, and 2 cycle samples) was dissolved in 300 μL of 1× PBS, and the 300 μL was applied to immobilized receptors with a flow rate of 50 μL/min at 25°C and pH 6.0. After each measurement, the surface of the sensor chip was regenerated using phosphoric acid buffer.

## Results

### Induction and Growth of Axillary Buds by Bottom Stem Cut

*Agrobacterium*-mediated tobacco plant transformation was conducted to generate transgenic plants expressing the anti-colorectal cancer mAb CO17-1A (Ko et al., 2005; So et al., 2012). The seeds of T_2_ transgenic plants were obtained by consecutive self-fertilization of the T_0_ and T_1_ plants. Two well-expanded true leaves appeared in the plantlets 21 days after sowing of T_2_ seeds. The growth period of the T_2_ plants until flower formation was around 100 days (**Figures 1A,B**). The lateral shoot was induced after retaining the root system by cutting the bottom stem of transgenic plants expressing the anti-colorectal cancer mAb CO17-1A (**Figure 1A**). Only a single lateral shoot was left to grow until just before the floral organ formation (**Figure 1A**). The bottom stem cutting was conducted in 2 cycles (**Figure 1A**). However, the growth period of the axillary shoot to the flowering stage from the cut stem was 30 and 35 days in the 1 and 2 cycles (**Figure 1B**). Overall, the growth period of the lateral shoot in 1 and 2 cycles was almost three times shorter compared to that of the primary shoot from the seedlings (0 cycle).

### Existence of mAb CO17-1A HC and LC Genes in 0, 1, and 2 Cycle Plants

The PCR analysis was conducted to confirm the existence of HC and LC genes of mAb CO17-1A in the leaf and stem tissues from top, middle, and base stem portions in 0, 1, and 2 cycles (**Figure 1C**). The HC and LC genes existed in all the portions of leaf and stem tissues of lateral shoot in all the cycles (**Figure 1C**). No HC or LC gene was amplified in the samples from the non-transgenic (NT) plants.

### HC and LC Protein Levels of mAb CO17-1A in the Leaf and Stem Tissues from Top, Middle, and Base Portions Through Recycling

The changes in HC and LC protein levels in top, middle, and basal leaves and stems in 0, 1, and 2 cycles were investigated by western blotting (**Figures 1D,E**). In the leaf tissue, the HC and LC protein levels were stable over the cycles (**Figure 1D** left and right panels, respectively). In the top leaves, the HC levels slightly increased with the cycles. The LC levels were steady over the cycles. In the stem tissue, the HC and LC levels were stable over the cycles (**Figure 1E** left and right panels, respectively). In the basal stems, the HC levels slightly decreased over the cycles (**Figure 1E** left panel). Overall, the HC and LC protein levels were similar in the samples from all the portions of stem through the cycles.

### Glycan Analysis of mAb^P^ CO17-1A Purified from Leaf and Stem of 0, 1, and 2 Cycle Plants

**FIGURE 2:**
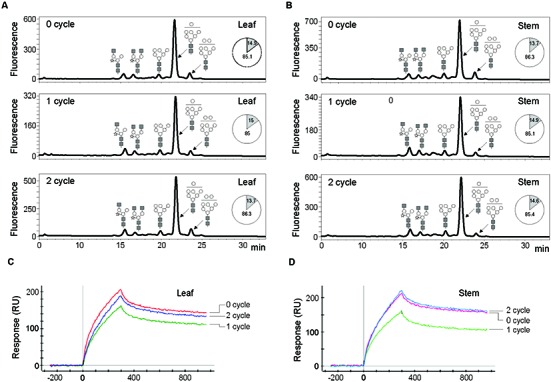
**Glycosylation and function analyses of mAb CO17-1A protein purified from leaves and stem of 0, 1, and 2 cycle plants. (A,B)** Profiles of *N*-glycan from mAb^P^ CO17-1A were analyzed using high performance liquid chromatography of the 2-AB labeled glycans. **(A)** Glycan structure profiles of mAb^P^ CO17-1A purified from leaf of 0, 1, and 2 cycle plants. **(B)** Glycan structure profiles of mAb^P^ CO17-1A purified from stem of 0, 1, and 2 cycle plants. GlcNAc, mannose, and xylose are depicted using black square, white circle, and white star, respectively. The ratios of oligomannose (white) and plant-specific (gray) glycans of mAb^P^ CO17-1A in leaves and stem of 0, 1, and 2 cycle plants were shown in a pie chart. Binding affinity of mAb CO17-1A purified from leaves **(C)** and stem **(D)** of 0, 1, and 2 cycle plants to GA733 antigen using surface plasmon resonance (SPR). Purified mAb^P^ CO17-1AK from the leaves of 0, 1, and 2 cycle plants was incubated with the GA733 adsorbed biochip **(C)**. Purified mAb^P^ CO17-1A from the stem of 0, 1, and 2 cycle plants was incubated with the GA733 adsorbed biochip **(D)**.

The *N*-glycans of mAb^P^ CO17-1A purified from the leaf and stem of 0, 1, and 2 cycle plants were analyzed by HPLC. The glycosylation patterns were analyzed in the leaf and stem (0, 1, and 2 cycles) tissues (**Figures 2A,B**). The glycan profile of leaf and stem from plants (0, 1 and 2 cycles) was similar and showed a high mannose-type glycan structure profile. The percentages (%) of oligomannose glycan in leaf and stem were ~15 and ~13.7–14.9, respectively, through the cycles (**Figures 2A,B**). The glycan structure profiles of mAb^P^ CO17-1A in 0, 1, and 2 cycle plant leaves and stems were similar.

### SPR Analysis of mAb CO17-1A Purified from Leaf and Stem of 0, 1, and 2 Cycle Plants

SDS-PAGE analysis was performed to identify the HC and LC of the purified mAb CO17-1A in the plant leaf and stem samples obtained in each cycle (0, 1, and 2; Data not shown). The purified mAb CO17-1A from 0, 1, and 2 cycle plants showed the same band sizes for HC and LC. Although the cycle number increased, there was no change in the quality of mAb CO17-1A, which remained undegraded. Expression and purity of mAb CO17-1A in stems was also confirmed in 0, 1, and 2 cycle plants. mAb^P^ CO17-1A purified from the leaf and stem samples of 0, 1 and 2 cycle plants were compared for their binding activities (**Figures 2C,D**). All the mAb^P^ CO17-1A purified from leaf and stem tissues collected in different cycles showed relatively similar interaction with the antigen GA733-Fc using SPR (**Figures 2C,D**). The binding affinities of the mAb CO17-1A purified from leaf and stem samples collected from different cycle plants were similar except for the 2 cycle where the mAb CO17-1A purified from stem (**Figure 2D**) showed slightly higher affinity for the antigen than the mAb purified from the leaf (**Figure 2C**). When the binding activities of mAb CO17-1A purified from the leaf and stem samples were compared among the cycles, the 1 cycle showed slightly lower affinity than the 0 and 2 cycles (**Figures 2C,D**). In general, however, the peaks of mAb CO17-A from both the leaf and stem samples were similar between the 0 and 2 cycles.

## Discussion

In the present study, we demonstrate that fresh stem and leaves regrow from axillary buds after cutting of the stem. The lateral shoots, thus generated, could stably express functional anti-colorectal cancer therapeutic antibody, mAb CO17-1A recombinant protein, without alterations in its glycosylation pattern.

The lateral shoots emerge from axillary meristems when the apical dominance is removed (Leyser, 2003). In the present study, the plants (1 and 2 cycles), induced to produce lateral branches from the axillary buds, grew faster (~30–35 days) to their full size and flowered than the plants grown from the seedlings (0 cycle), which required ~100 days to reach their full size.

In fact, the use of plant expression systems has been limited due to longer growth period to obtain a full-sized plant with high biomass (Hood et al., 2002; Horn et al., 2004; Teli and Timko, 2004). In our previous study, fully grown *Nicotiana tabacum* plants started to form the floral organs at 12 weeks after sowing (Lim et al., 2015), which is much longer than *N. benthamiana* (7 weeks; Conley et al., 2011). *N. benthamiana* is another host plant for production of recombinant proteins such as vaccine and antibody, and has been established for their transient expression (Gomez et al., 2013; Li et al., 2016). *N. benthamiana* needs to be transfected every time with expression vector inoculums to produce recombinant proteins. In addition, their left over biomass should be properly discarded for avoiding contamination, and the transfected plant can not be regrown for further transfection usage. Thus, the transgenic plant regrowth by axillary shoot induction with less than 4 weeks appears to be an easy method to quickly increase the full biomass for production of recombinant proteins even under limitations of space. Our results suggest that axillary bud induction from the base stem with root could be used in molecular biofarming strategies to overcome the constraints of space and time.

The existence of HC and LC genes in both the leaves and stem generated from the axillary buds during the regrowth cycles was confirmed using PCR analysis, which revealed that the genes were present in the top, middle, and basal portions of the leaves and stem of the plants (0, 1, and 2 cycles) without any deletion.

The expression of HC and LC of mAb CO17-1A in the leaves and stem from the top, middle, and basal positions obtained from 0, 1, and 2 cycle plants was confirmed by western blot. The HC and LC expression rates were not significantly different among the samples.

The mAb CO17-1A purified from the plants had a mainly oligomannose structure profiles because of the C-terminus KDEL signal tagging of HC for ER retention (So et al., 2012). The glycosylation profiles were unmodified in leaf and stem in samples from the 0, 1, and 2 cycles.

The mAb CO17-1A purified from primary (0 cycle), secondary (1 cycle), and tertiary (2 cycle) plants showed similar binding affinity to the GA733 antigen in SPR analysis. Leaves and stem from the 1 cycle showed slightly lower binding activity than those from the 0 and 2 cycle plants. However, it is speculated that the slight fluctuation in the binding activity was due to the variation in sample preparation, and not due to an actual loss in the activity. The binding activities of mAb CO17-1A purified from both the leaves and stem of plants from the same cycle were similar. The results present notable evidence that plant recycling can be applied for efficient biomass enhancement without any variation in expression and function of the recombinant anti-cancer therapeutic mAb. The rapid plant regrowth using by the existing lateral buds in stem attached to the root is possible for the plant biomass production in a limited space.

Taken together, the leaf and stem of the secondary and tertiary cycles of plant growth (1 and 2 cycles) had similar mAb CO17-1A expression rate, and the antigen affinity as well as glycan structure profile of the purified mAb were comparable to the purified mAb samples obtained from the primary plant growth (0 cycle). This study shows that novel recycling plant system by using regrowth from axillary buds can effectively circumvent the space and time limitations for cultivation of plants. The strategy of recycling plant production could be exploited for obtaining increased transgenic plant biomass in less time and could be useful for producing highly valuable recombinant proteins for varied use.

## Author Contributions

KK and D-SK conceived and designed the experiments. D-SK and IS performed the experiments. KK, D-SK, and IS analyzed the data. D-SK and JK contributed reagents/materials/analysis tools. KK and D-SK wrote the paper. All of the authors carefully checked and approved this version of the manuscript.

## Conflict of Interest Statement

The authors declare that the research was conducted in the absence of any commercial or financial relationships that could be construed as a potential conflict of interest.
